# An exceptional case of huge myxofibrosarcoma of the pericardium

**DOI:** 10.1093/jscr/rjaf270

**Published:** 2025-05-02

**Authors:** Mehdi Laaroussi, Mohammed Tribak, Abdel Malick Idrissa, Abdramane Maiga, Adama Boite, Briki Jihad, Chakib Benlafqih, Rochde Sayah, Mohamed Cherti, Mohammed Laaroussi

**Affiliations:** Cardiology “B” Department, Ibn Sina University and Hospital Centre, Mohammed V Souissi University, Rabat 10800, Morocco; Cardiovascular Surgery “A” Department, Ibn Sina University and Hospital Centre, Mohammed V Souissi University, Rabat 10800, Morocco; Cardiovascular Surgery “A” Department, Ibn Sina University and Hospital Centre, Mohammed V Souissi University, Rabat 10800, Morocco; Cardiovascular Surgery “A” Department, Ibn Sina University and Hospital Centre, Mohammed V Souissi University, Rabat 10800, Morocco; Cardiovascular Surgery “A” Department, Ibn Sina University and Hospital Centre, Mohammed V Souissi University, Rabat 10800, Morocco; Cardiovascular Surgery “A” Department, Ibn Sina University and Hospital Centre, Mohammed V Souissi University, Rabat 10800, Morocco; Cardiovascular Surgery “A” Department, Ibn Sina University and Hospital Centre, Mohammed V Souissi University, Rabat 10800, Morocco; Cardiovascular Surgery “A” Department, Ibn Sina University and Hospital Centre, Mohammed V Souissi University, Rabat 10800, Morocco; Cardiology “B” Department, Ibn Sina University and Hospital Centre, Mohammed V Souissi University, Rabat 10800, Morocco; Cardiovascular Surgery “A” Department, Ibn Sina University and Hospital Centre, Mohammed V Souissi University, Rabat 10800, Morocco

**Keywords:** myxofibrosarcoma pericardium, cardiac malignancy, cardiac surgery

## Abstract

Primary cardiac myxofibrosarcoma is a rare malignant tumour usually found in the left atrium. Its pericardial location is extremely rare, and its management is mainly based on surgery. We report a historical case of myxofibrosarcoma of the pericardium weighing 4200 g.

## Introduction

Primary cardiac tumours are rare, accounting for between 0.001% and 0.03% of cases observed in autopsy series [1]. Of these, malignant tumours account for a quarter, of which sarcoma is the most common [2]. In the pericardium, primary tumours are even rarer, with a prevalence of 0.001% to 0.007% [3], mesothelioma being the most common malignant tumour [4]. Pericardial myxofibrosarcoma is an extremely rare tumour usually found in the left atrium (LA). We report a case of myxofibrosarcoma that is exceptional in terms of its nature, location and size.

## Case presentation

A 55-year-old man was admitted to emergency for history of chest pain associated with New York Heart Association stage III dyspnoea that had been ongoing for 4 months. He had no past medical history. Clinical examination revealed a hemodynamically stable patient with pulsed oxygen saturation (SpO_2_) of 90% on room air.

**Figure 1:**
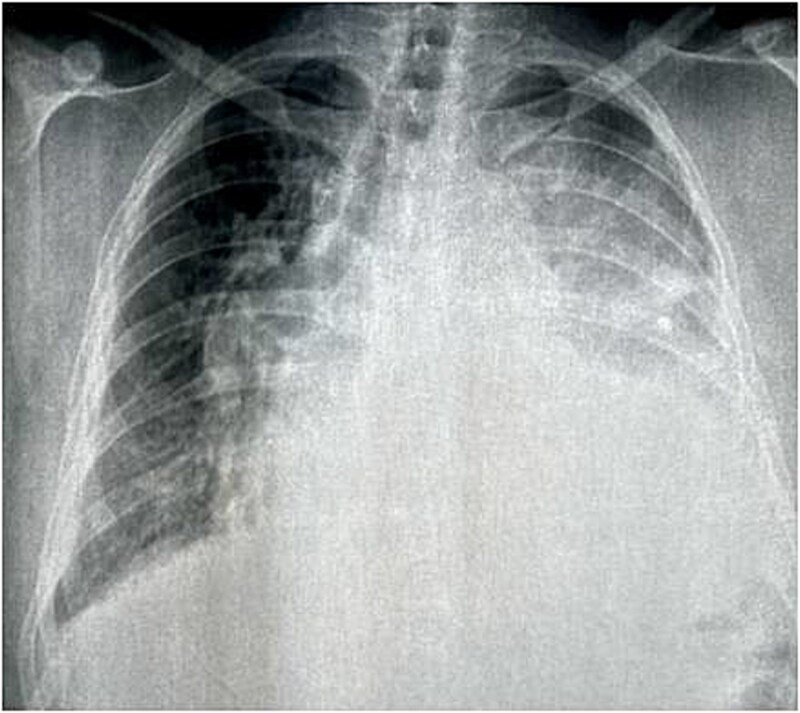
Chest X-ray showed a left opacity that encompassed the cardiac outline and displaced the trachea and stem bronchi on the right.

Chest X-ray revealed a left opacity that encompassed the cardiac outline and displaced the trachea and stem bronchi on the right (Fig. 1).

Transthoracic echocardiography (TTE) revealed a large pericardial effusion with a maximum of 40 mm latero-LA and 11 mm latero-right ventricle, good biventricular function with a left ventricular ejection fraction of 58% and no valvular disease.

**Figure 2:**
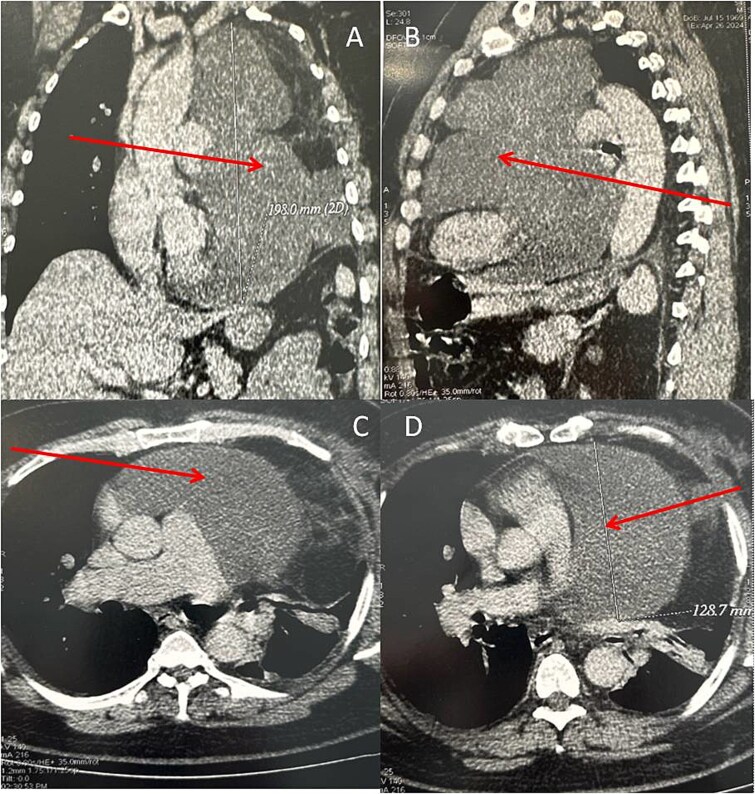
Chest CT scan (A: coronal section, B: sagittal section, C and D: axial section) in spiral mode with injection of contrast agent showing a giant pericardial mass (arrows).

Because of the patient’s very poor echogenicity, TTE was unable to provide useful information, unlike thoracic computed tomography (CT), which revealed a mass of fluid density and homogeneous content, developed in the left pericardium and measuring 185 mm in height, 120 mm in transverse diameter and 140 mm  in antero-posterior diameter. This mass compressed the left lung, which was reduced in volume, and displaced the heart chambers to the right (Fig. 2).

**Figure 3:**
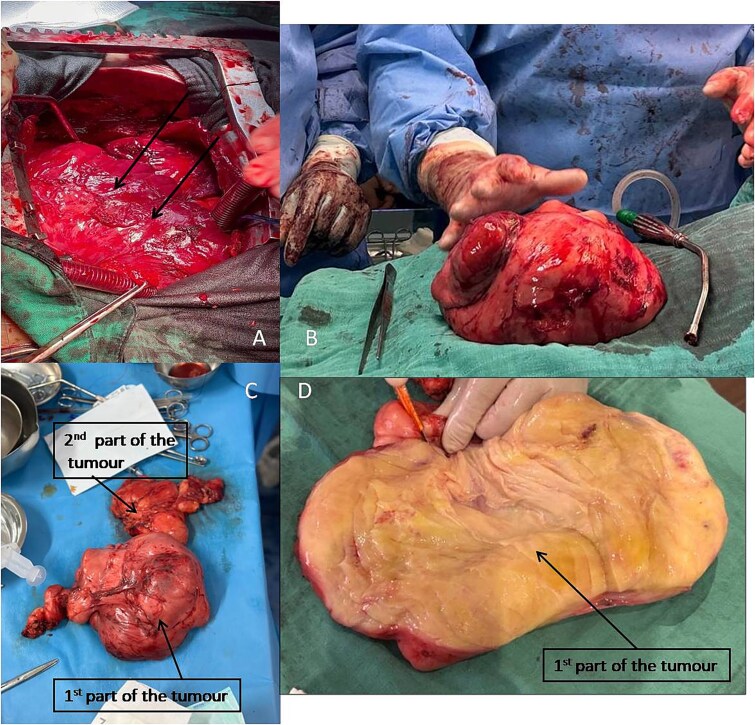
(A) Intraoperative image showing the tumour (arrow). (B) First part of the tumour removed, compared macroscopically with a hand. (C) Complete tumour removed in two separate parts. (D) First part of the tumour dissected to reveal internal contents.

The patient was admitted to the operating room for pericardial drainage and tumorectomy by full sternotomy under cardiopulmonary bypass (CPB), which resulted in the resection of a reddish tumour with a firm consistency and irregular contours, weighing 4200 g and a second tumour of the same nature, adherent to the pleura and weighing 300 g (Fig. 3).

Hemostasis was performed at the tumour resection sites, which showed excessive bleeding. The patient was discharged on bypass after 170 minutes of assistance under positive inotropic drugs. Specimens were sent for histological study.

The immediate postoperative follow-up was marked by hemodynamic instability refractory to intensive care measures, complicated by cardiogenic shock with a fatal outcome.

**Figure 4:**
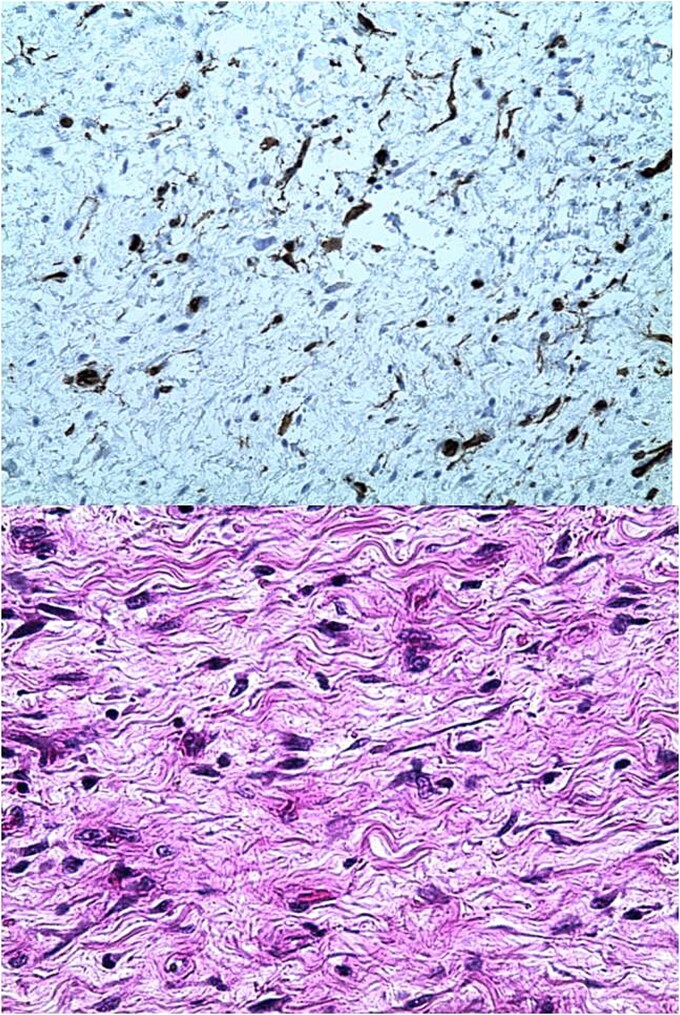
Images showing histological and immunohistochemical sections of the removed tumour.

Histological and immunohistochemical studies were consistent with low-grade myxofibrosarcoma (Fig. 4).

## Discussion

Cardiac sarcomas account for 20% of primary malignant cardiac tumours, of which myxofibrosarcomas are an extremely rare entity. The World Health Organization classification of cardiac tumours defines myxofibrosarcoma as a malignant tumour composed of fibroblasts with variable amounts of intercellular collagen and abundant myxoid stroma [5]. Its preferred location in the heart is the LA [6]; pericardial location is exceptional, and is mainly associated with tamponade.

Consultation of the PubMed database shows only one case of myxofibrosarcoma with simultaneous involvement of the LA and the pericardium reported by Azuma *et al.* [6]. On the other hand, Soltani *et al.* [2] consulted the PubMed, Medline and Web of science databases and found no cases of myxofibrosarcoma of the pericardium between 2018 and 2023. Similar studies have not found a pericardial location [5, 7]. This is to highlight the extreme rarity of pericardial localization of myxofibrosarcoma, which consequently makes the case we are reporting an exceptional one. It is in fact a myxofibrosarcoma that exclusively affects the pericardium with pleural extension.

This case is unusual in that it measures 185 mm in height, 120 mm in transverse diameter, and 140 mm in anteroposterior diameter, with a historical weight of 4200 g. There is no cardiac tumour, either intracavitary or pericardial, with these measurements. Soltani *et al.* reported a case of myxofibrosarcoma of the LA measuring 80/60/20 mm [2]. Kwon *et al.* reported a case of a rapidly growing undifferentiated sarcoma of the pericardium, which grew from 37/95 to 80/150 mm in 3 months and was also fatal to the patient in the setting of heart failure and arrhythmias [8]. Other authors have reported pericardial lipomas measuring 120/120/80 and 40/100 mm, respectively [9, 10]. All these reported cases present relatively exceptional measurements, but they remain smaller than those of the case reported here.

Management of primary cardiac tumours is not codified and is essentially based on incomplete or complete surgical resection, under CPB or beating heart, with or without chemotherapy and/or radiotherapy. In the series by Sun *et al.* [5], of the 31 cases collected, 26 underwent resection surgery, and of these 21, 11 received adjuvant treatment: chemotherapy (5 cases), radiotherapy (4 cases), and combined chemo-radiotherapy (2 cases). The outcome revealed tumour recurrence in nine patients, metastases in four patients, and no evidence of disease in eight patients. The rates of local recurrence and metastasis were 42.9% and 19%, respectively [5].

For survival, a threshold diameter of >40 mm is statistically significant. Our patient underwent complete resection of myxofibrosarcoma of the pericardium and pleura, but unfortunately died postoperatively due to bleeding complications and cardiogenic shock.

## Conclusion

Primary myxofibrosarcoma of the pericardium is an extremely rare malignant tumour. We report a historical case of myxofibrosarcoma of the pericardium with a fatal postoperative outcome. Earlier diagnosis is crucial for appropriate management and a favorable outcome, allowing patients to benefit from complete surgical resection and radio-chemotherapy.
